# Long-term cilostazol treatment reduces gliovascular damage and memory impairment in a mouse model of chronic cerebral hypoperfusion

**DOI:** 10.1038/s41598-017-04082-0

**Published:** 2017-06-27

**Authors:** Akihiro Kitamura, Yasmina Manso, Jessica Duncombe, James Searcy, Juraj Koudelka, Margaret Binnie, Scott Webster, Ross Lennen, Maurits Jansen, Ian Marshall, Masafumi Ihara, Raj N. Kalaria, Karen Horsburgh

**Affiliations:** 1 University of Edinburgh, Edinburgh, UK; 2 National Cerebral and Cardiovascular Center, Osaka, Japan; 3 Institute of Neuroscience, Newcastle University, Newcastle, UK

## Abstract

Chronic cerebral hypoperfusion is a major cause of age-related vascular cognitive impairment. A well-characterised mouse model has shown that hypoperfusion results in gliovascular and white matter damage and impaired spatial working memory. In this study, we assessed whether cilostazol, a phosphodiesterase III inhibitor, could protect against these changes. Adult, male C57Bl/6J mice were subjected to bilateral common carotid artery stenosis or a sham operation and fed normal or cilostazol diet for three months. Cilostazol treatment reduced the impairment in working memory and white matter function after hypoperfusion. Endothelial adhesion molecules and gliosis, increased after hypoperfusion, were ameliorated with cilostazol treatment. Interestingly, the improvement in working memory was closely correlated with reduced microglia and endothelial adhesion molecules. Further, the number of stroke lesions after hypoperfusion was reduced in the cilostazol-treated group. Altogether cilostazol showed potential to ameliorate the gliovascular damage and working memory impairments after hypoperfusion possibly via endothelial protection supporting its potential use in the treatment of vascular cognitive impairment.

## Introduction

Vascular cognitive impairment (VCI) is a spectrum of age-related cognitive decline caused by vascular factors such as hypertension, atherosclerosis and diabetes^1^. One of the most common forms of VCI is small vessel disease which is associated with diffuse white matter injury, subcortical lacunar infarcts and microbleeds. The mechanisms leading to VCI are ill-defined but disruption of the interplay between cells within the neuro-glio-vascular unit have been shown such as impaired vascular haemodynamics, endothelial dysfunction and compromise of the blood brain barrier (BBB). Another key and common mechanism linked to VCI is chronic cerebral hypoperfusion which is induced by vessel narrowing and endothelial dysfunction. Cerebral hypoperfusion is now emerging as a major contributor to cognitive decline^2^.

Several animal models have been developed to investigate the mechanisms by which chronic hypoperfusion may lead to VCI. Of these, an experimental mouse model of chronic cerebral hypoperfusion, induced by bilateral carotid stenosis, is considered to be the most clinically relevant and has been widely used to probe mechanisms related to VCI. In this model, modest reductions in cerebral blood flow, akin to that observed in clinical VCI, can be reproduced using microcoils that when applied to both common carotid arteries restrict blood flow to the forebrain in mice. We have shown in this mouse model that cerebral hypoperfusion results in early (days to weeks) disruption of axon-glial integrity, myelin damage, microglial proliferation and spatial memory impairment^3, 4^ with subsequent (3–6 months) gliovascular unit damage, BBB disruption and small vessel changes^5^. This tractable model provides a basis to test potential treatment strategies for VCI.

To date there remains no effective treatments for VCI and instead current therapies are targeted to ameliorate the cognitive impairments via anti-cholinesterase approaches. Current drug developments are focussed on strategies to improve vascular health and the gliovascular unit^6, 7^. Since multiple mechanisms may be involved in VCI, drugs that have pleiotropic effects may be particularly useful. One such drug, cilostazol, a phosphodiesterase III inhibitor, is known to have neuro-glio-vascular protective effects through multiple mechanisms. Cilostazol is approved as an antiplatelet agent for the prevention and treatment of cerebral infarction and peripheral arterial disease. Cilostazol increases cAMP in vascular cells and can exert multiple beneficial effects on the vasculature such as endothelial protection^8, 9^, maintenance of microvascular integrity^10^, vasodilation, anti-oxidation, anti-inflammation, regulation of smooth muscle cells^11^ and increase in cerebral haemodynamics^12^. There is now emerging evidence to suggest cilostazol may have beneficial effects in models of VCI and protect against white matter damage and cognitive impairment. However, to date the majority of these studies have focussed on the short-term effects of cilostazol over weeks^13–16^. The present study assessed whether cilostazol could protect long-term against the degenerative changes within the gliovascular unit and cognitive impairment induced by chronic cerebral hypoperfusion and whether it could exert beneficial effects on microvascular inflammation.

## Results

### Cilostazol had modest effects on spatial working memory after hypoperfusion

**Figure 1:**
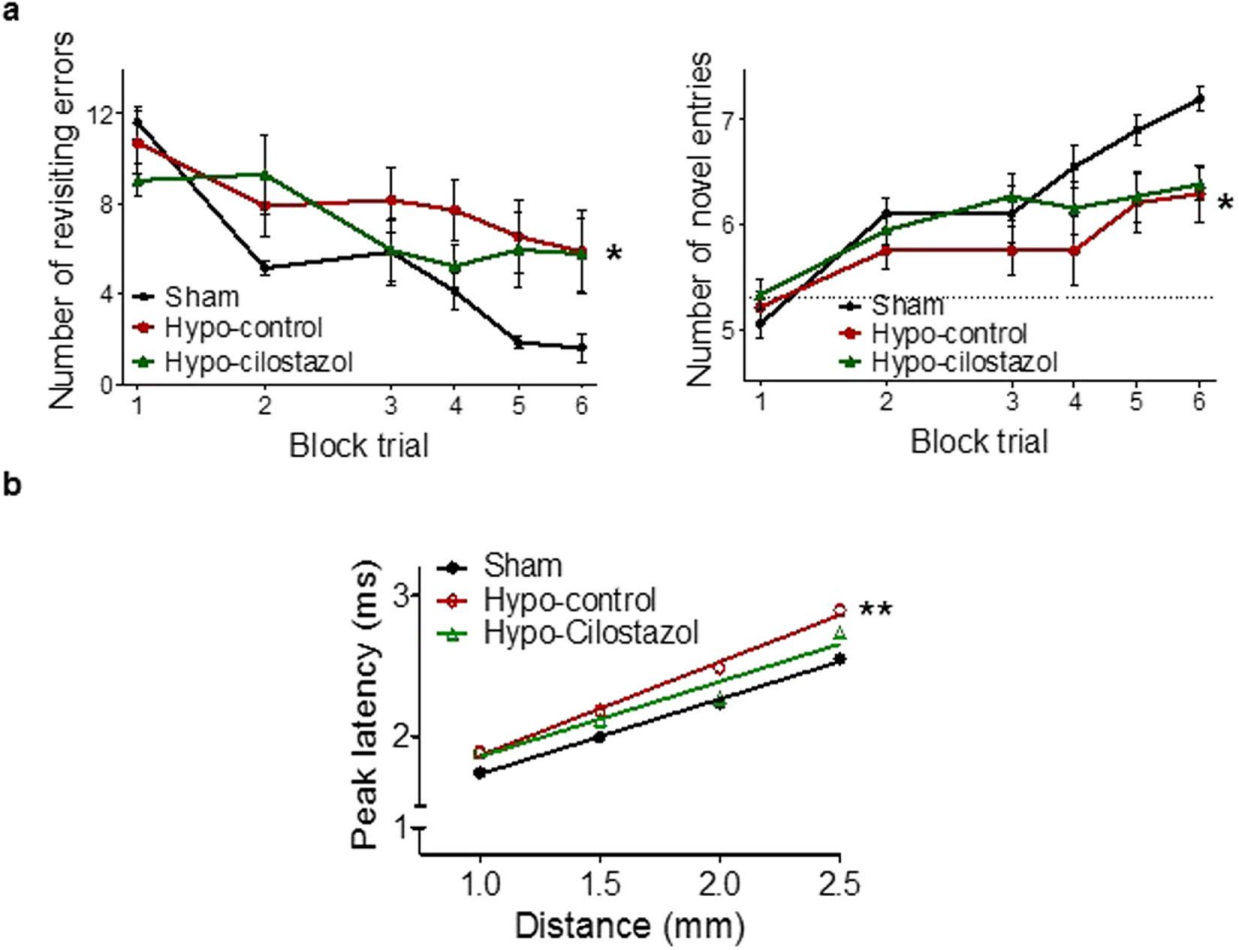
Cilostazol improved spatial working memory and white matter function: (**a**) Spatial working memory was assessed using an 8-arm radial arm maze. Statistical analysis of the number of revisiting errors and novel entries indicated an overall significant difference between the 3 groups (*F*_(2, 34)_ = 5.169; *p* = 0.029 and *F*_(2, 34)_ = 5.718; *p* = 0.022, respectively). Hypoperfused-control mice exhibited significantly more revisiting errors and less novel entries than the sham operated mice (*p* < 0.05 and *p* < 0.05), while hypoperfused-cilostazol mice showed a learning process similar to that of sham mice before they plateaued. Data represents the average of 4 trials for block 1 and 2 and the average of 2 trials for blocks 3–6. (**b**) In the corpus callosum, evoked compound actions potentials (CAPs) were recorded at various distances from the stimulating electrode. Peak latencies of CAPs were significantly different between the three groups (*F*_(2,12)_ = 14.78; *p* = 0.0048). The peak latency of CAPs in hypoperfused-control mice was overall significantly increased compared to sham mice (*p* < 0.01) but this was ameliorated with cilostazol treatment. Data represents mean ± SEM. **p < 0.01, Hypoperfused-control vs Sham.

Spatial working memory has been shown to be selectively impaired in response to hypoperfusion^3, 17^. Similarly, the hypoperfused-control mice showed a clear impairment in this task and committed more revisiting errors (Fig. 1a) and performed less novel entries (Fig. 1a) than the sham mice throughout the test. Noteworthy, the hypoperfused-cilostazol mice showed a learning process similar to shams up to block trial 4 (day 12 of test) before they plateaued (Fig. 1a) suggesting that the spatial memory impairment, whilst reduced, was not completely ameliorated with cilostazol treatment. Statistical analysis of the number of revisiting errors and novel entries indicated an overall significant difference between the 3 groups (*F*_(2,34)_ = 5.169; *p* = 0.029 and *F*_(2,34)_ = 5.718; *p* = 0.022, respectively). Post-hoc analysis indicated that there was a significant difference in the number of revisiting errors and novel entries between the hypoperfused-control and the sham group (*p* < 0.05), but there was no difference between the hypoperfused-cilostazol and sham group (*p* > 0.05). This data suggests that the chronic treatment with cilostazol has modest beneficial effects on spatial working memory after long term hypoperfusion.

### Cilostazol improved white matter function

We have previously demonstrated that chronic cerebral hypoperfusion causes diffuse white matter changes^3^. To build on this work, we determined whether the functional integrity of white matter was altered in response to hypoperfusion and if this was ameliorated by cilostazol. Peak latencies of compound action potentials (CAPs) were significantly different between the three groups (*F*_(2, 12)_ = 14.78; *p* = 0.0048) Post-hoc analysis indicated that the peak latency of CAPs in hypoperfused-control mice were significantly increased compared to shams (*p* < 0.01). However, the peak latencies of CAPs in the hypoperfused-cilostazol group were not significantly different to those of sham mice (*p* > 0.05) (Fig. 1b). These results suggest that hypoperfusion significantly impedes the conduction velocity of myelinated fibers and that cilostazol treatment is able to reduce this deficit.

### Cilostazol did not significantly restore white matter integrity

**Figure 2:**
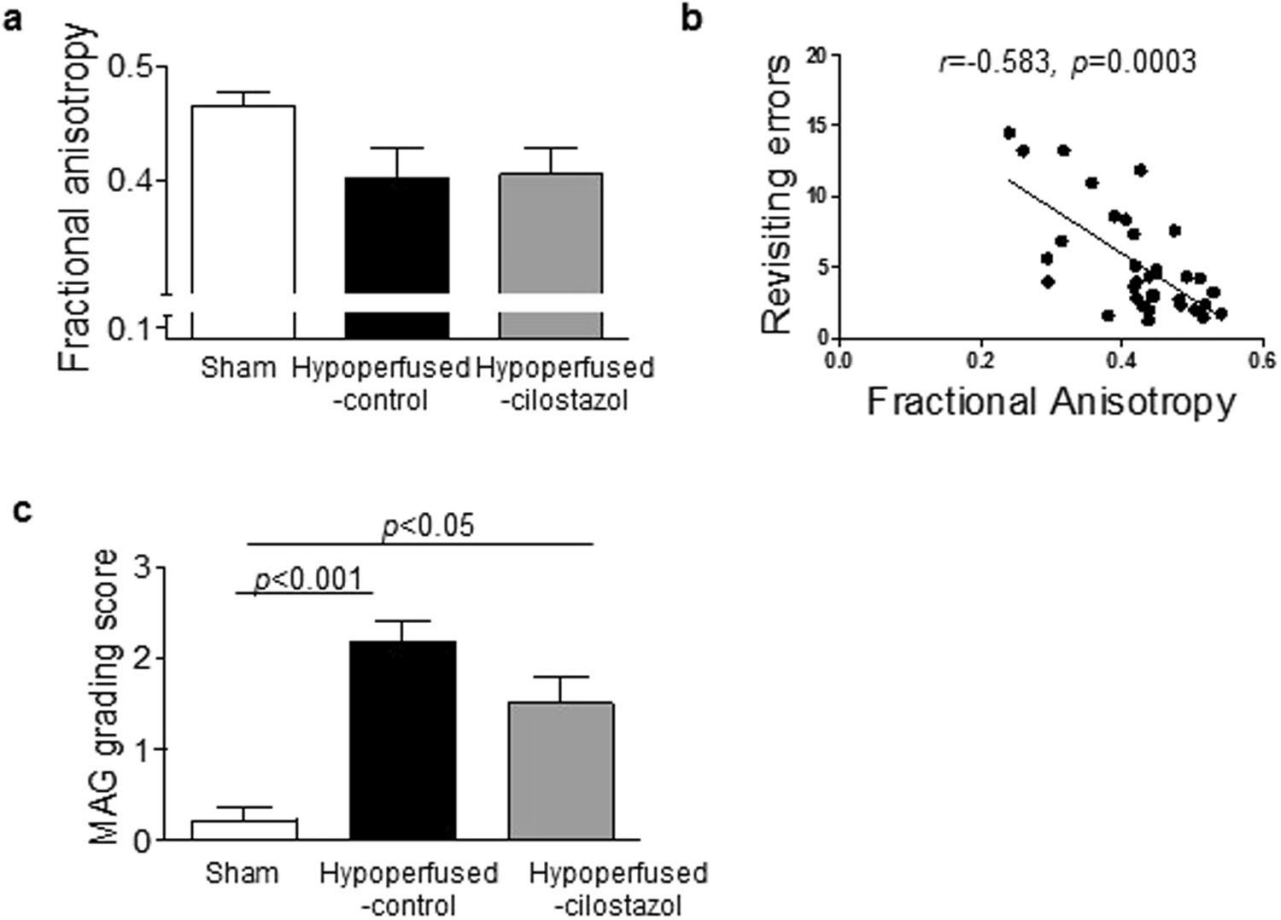
Cilostazol did not significantly restore white matter integrity. (**a**) There was no significant difference in Fractional Anisotropy (FA) in the corpus callosum between the three groups. (**b**) A significant relation between FA and revisiting errors was determined (Spearman *r* = −0.583, *p* = 0.0003). (**c**) MAG grading, as an index of breakdown in axon-glial integrity, was significantly increased after hypoperfusion, but no protective effect of cilostazol could be observed. Data represents mean ± SEM. **p < 0.001, Hypoperfused-control vs Sham; **p < 0.05, Hypoperfused-Cilostazol vs Sham.

Since white matter function was found to be compromised with hypoperfusion and restored with cilostazol treatment, we next evaluated white matter integrity using diffusion tensor imagingimaging (DTI) and pathological analysis (myelin associated glycoprotein, MAG, immunostaining). There was no significant difference in fractional anisotropy (FA) in the corpus callosum between the groups (*F*_(2,33)_ = 2.513; *p* = 0.097) (Fig. 2a). However it was noted that there was a modest reduction in FA between the sham and hypoperfused-control groups (0.47 ± 0.01 vs. 0.40 ± 0.03), consistent with a previous report^18^. Cilostazol treatment did not have any effect on FA (0.40 ± 0.02). Interestingly, there was a significant negative correlation between FA and the number revisiting errors made on the radial arm maze test with mice committing more errors having lower FA values which are indicative of a more disrupted white matter architecture (Fig. 2b) (Spearman *r* = −0.583, *p* = 0.0003).

There was a significant difference in MAG staining between the three groups (*p* = 0.0003). Post-hoc analysis indicated that there was a significant loss of axon-glial integrity (identified by MAG immunostaining) in the hypoperfused-control compared to the sham mice in the corpus callosum (*p* < 0.001). However, the loss of axon-glial integrity was also found to be significantly different in the hypoperfused-cilostazol mice as compared to sham mice (*p* < 0.05) (Fig. 2c) indicating that cilostazol was unable to improve axon-glial disruption after hypoperfusion.

### Cilostazol improved cellular neuroinflammation

**Figure 3:**
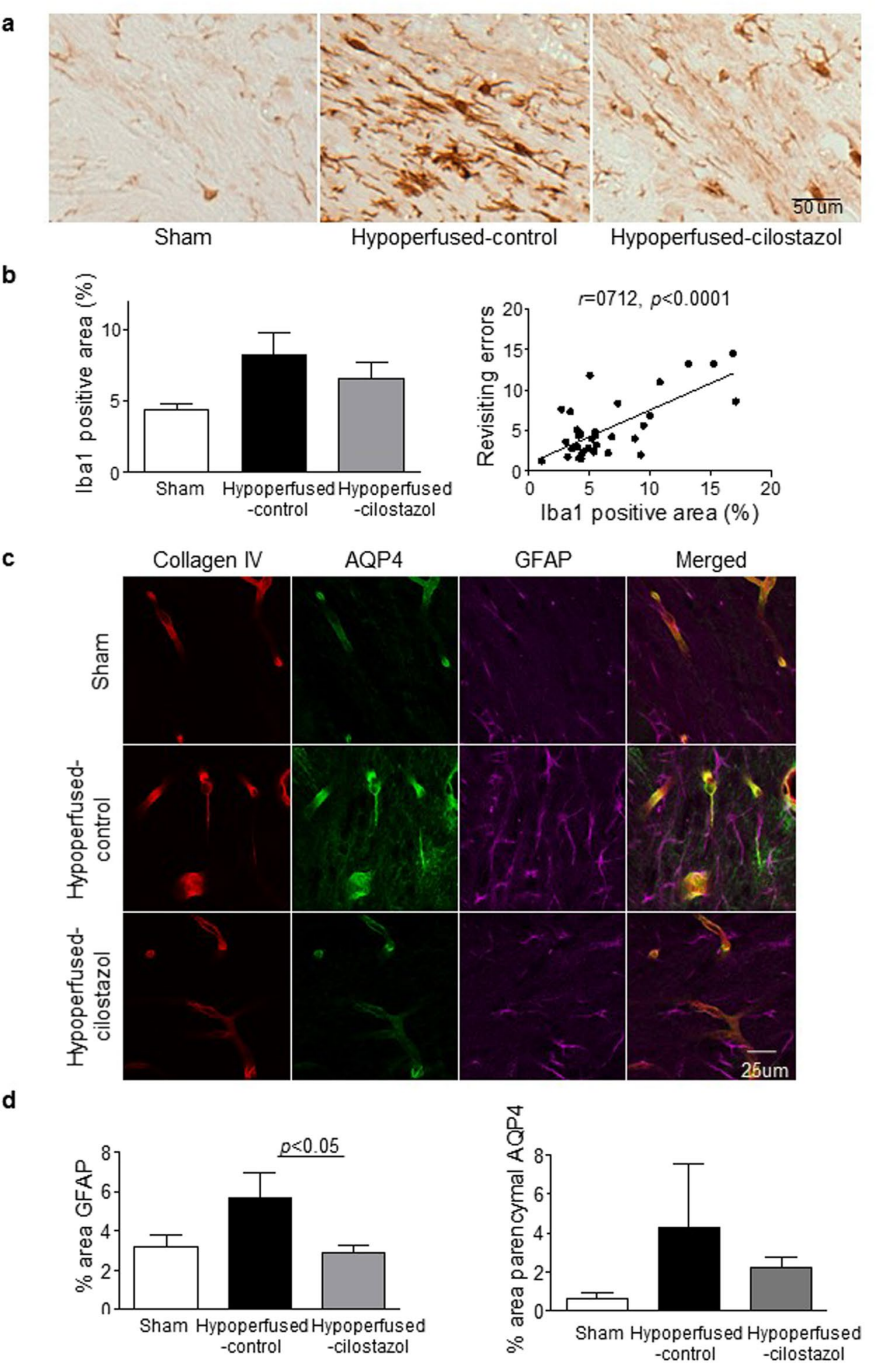
Cilostazol reduced the extent of gliosis. (**a**) Representative images of Iba1 staining in the corpus callosum. (**b**) There was a trend towards an increase in Iba1 stained micoglia that was reduced with cilostazol after hypoperfusion. There was a robust association between microgliosis and the number of revisiting errors (Pearson *r* = 0.712, *p* < 0.0001). (**c**) Representative images of immunofluorescence for Collagen IV, AQP4 and GFAP in the corpus callosum. (**d**) There was an increase in the area of GFAP positive astroglia in the hypoperfused-control mice. There was a significant reduction with cilostazol treatment as compared to control hypoperfused. Astrocyte-endfoot displacement tended to be observed in the hypoperfused-control mice and less in the cilostazol treated mice. Data represents mean ± SEM. *p < 0.05, Hypoperfused-control vs Hypoperfused-cilostazol.

A prominent feature of hypoperfusion is a robust cellular neuroinflammatory response. Consistent with this, microglial activation (identified by the extent of Iba1 immunostaining) was increased in the corpus callosum after hypoperfusion (Fig. 3a) but in this study we found no significant overall effect between the 3 groups (*F*_(2, 33)_ = 2.866; *p* = 0.072). However the results did indicate that there was an increase in the density of Iba1 positive staining following hypoperfusion which was lower with cilostazol treatment (8.26 ± 1.47 *vs* 6.61 ± 1.05) (Fig. 3b). Notably, there was a robust association between the extent of Iba1 staining in the corpus callosum and revisiting errors (Fig. 3b, Pearson *r* = 0.712, *p* < 0.0001), indicating that higher levels of cellular neuroinflammation correlate with poorer spatial working memory. Similarly, in the corpus callosum there was an overall difference in the extent of reactive astrogliosis (glial fibrillary acidic protein, GFAP, immunostaining) (*p* = 0.04). It was found that GFAP immunostaining was significantly reduced after hypoperfusion with cilostazol treatment (*p* < 0.05) (Fig. 3c and d).

To explore potential dynamic changes in gliovascular unit, alterations in the protein aquaporin 4 (AQP4) were investigated. AQP4 is a water channel typically confined to the endfeet of vascular associated astrocytes and is reported to maintain osmotic balance and to lose the polarization to the perivascular endfeet related to the development of microinfarcts^19, 20^. Mislocalisation of the water channel AQP4 (as measured by % area AQP4) was observed in the hypoperfused-control mice compared to sham mice consistent with our previous study^5^ and cilostazol tended to revert this redistribution (Sham; 0.67 ± 0.25, Hypoperfused-control; 4.31 ± 3.21, Hypoperfused-cilostazol; 2.20 ± 0.56, *p* = 0.12) (Fig. 3d).

### Cilostazol significantly suppressed endothelial adhesion molecule expression

**Figure 4:**
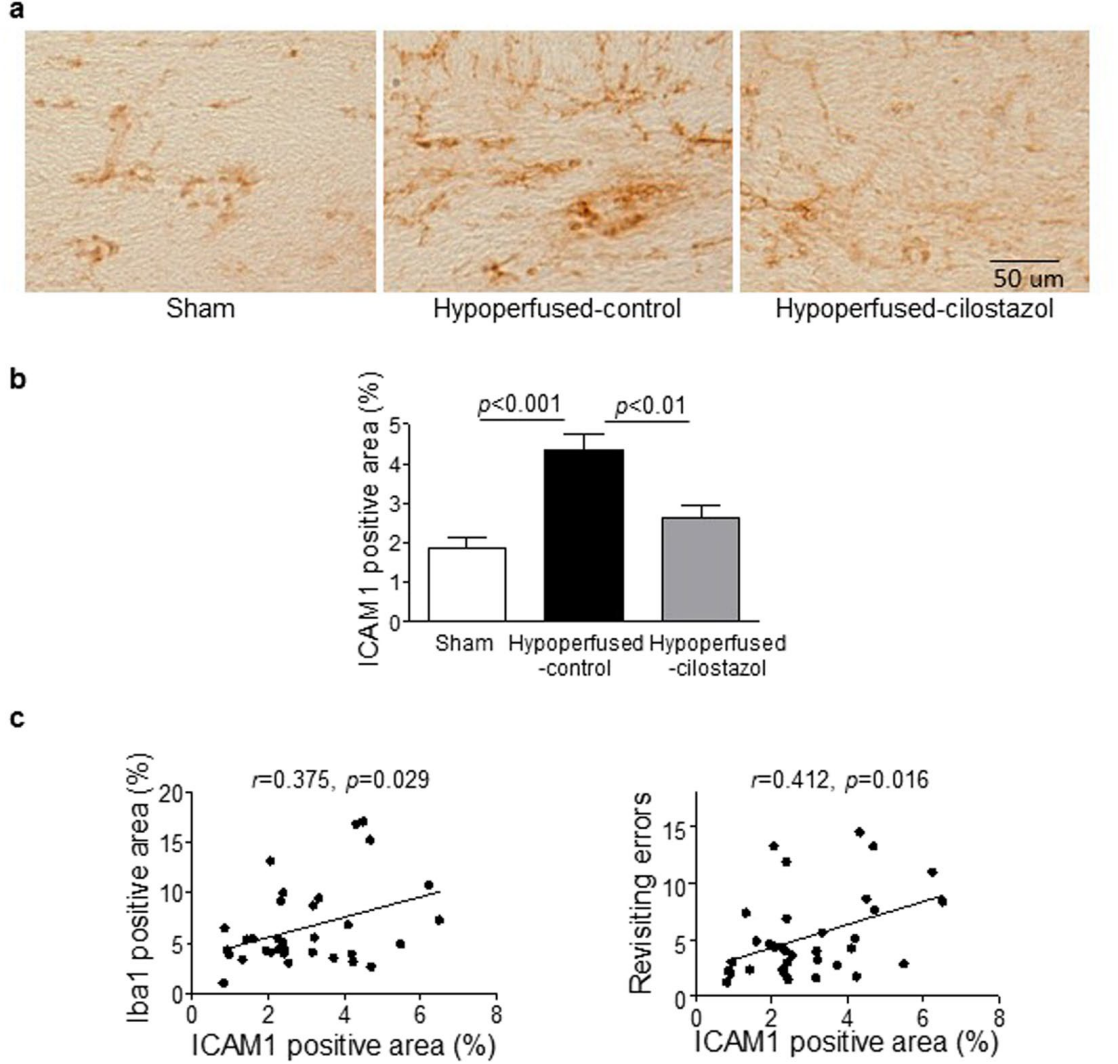
Cilostazol significantly suppressed endothelial adhesion molecule expression in the corpus callosum. (**a**) Representative images of intercellular adhesion molecule-1 (ICAM1) staining in the corpus callosum. (**b**) There was a significant difference in ICAM staining between the three groups (*F*_(2, 33)_ = 13.19; *p* < 0.0001). ICAM1 immunostaining was significantly greater in the hypoperfused-control group compared to the sham (*p* < 0.001) and this was reduced with cilostazol treatment (*p* < 0.01). (**c**) There was a significant association between ICAM1 positive areas and microgliosis (Pearson *r* = 0.375, *p* < 0.05), and the number of revisiting errors (Pearson *r* = 0.412, *p* < 0.05). Data represents mean ± SEM.

In order to elucidate the mechanism by which cilostazol reduces glial activation, we measured endothelial activation, previously shown to be linked to beneficial effects of cilostazol^8–10^. Intercellular adhesion molecule-1 (ICAM1), a marker of endothelial cell activation^21^, was investigated. There was a significant difference in ICAM between the three groups (*F*_(2, 33)_ = 13.19; *p* < 0.0001). A significant increase in ICAM1 immunostaining was determined in the corpus callosum in the hypoperfused-control group compared to both the sham and hypoperfused-cilostazol groups (*p* < 0.001 and *p* < 0.01) (Fig. 4a and b). Supportive of a close relation between endothelial cell activation, microgliosis and memory, a significant association between ICAM1 and Iba1 immunostaining was determined (Pearson *r* = 0.375, *p* = 0.029), and a significant association between ICAM1 and the number of revisiting errors (Pearson *r* = 0.412, *p* = 0.016) (Fig. 4c) was determined in the corpus callosum.

### Cilostazol reduced stroke lesions but did not affect baseline CBF

**Figure 5:**
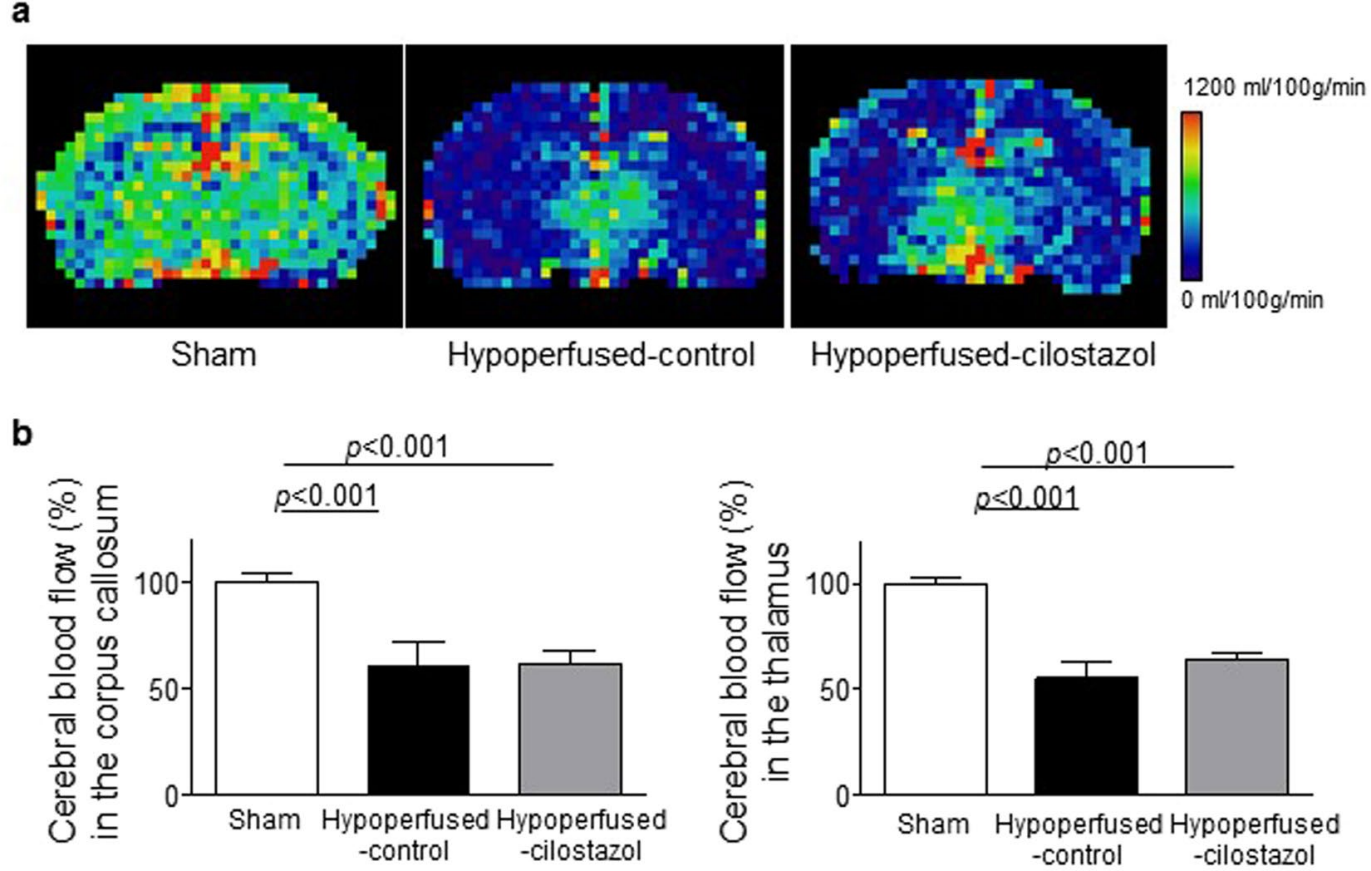
Cilostazol did not affect cerebral blood flow. (**a**) Representative images of ASL at the level of hippocampus. (**b**) There was an overall difference in CBF between the three groups in the corpus callosum (*F*_(2, 25)_ = 11.0; *p* = 0.0004) and thalamus (*F*_(2, 25)_ = 33.05; *p* < 0.0001). CBF was significantly reduced in hypoperfused-control and hypoperfused-cilostazol mice compared to sham mice in the corpus callosum (*p* < 0.001 and *p* < 0.001) and the thalamus (*p* < 0.001 and *p* < 0.001). Data represents mean ± SEM.

**Figure 6:**
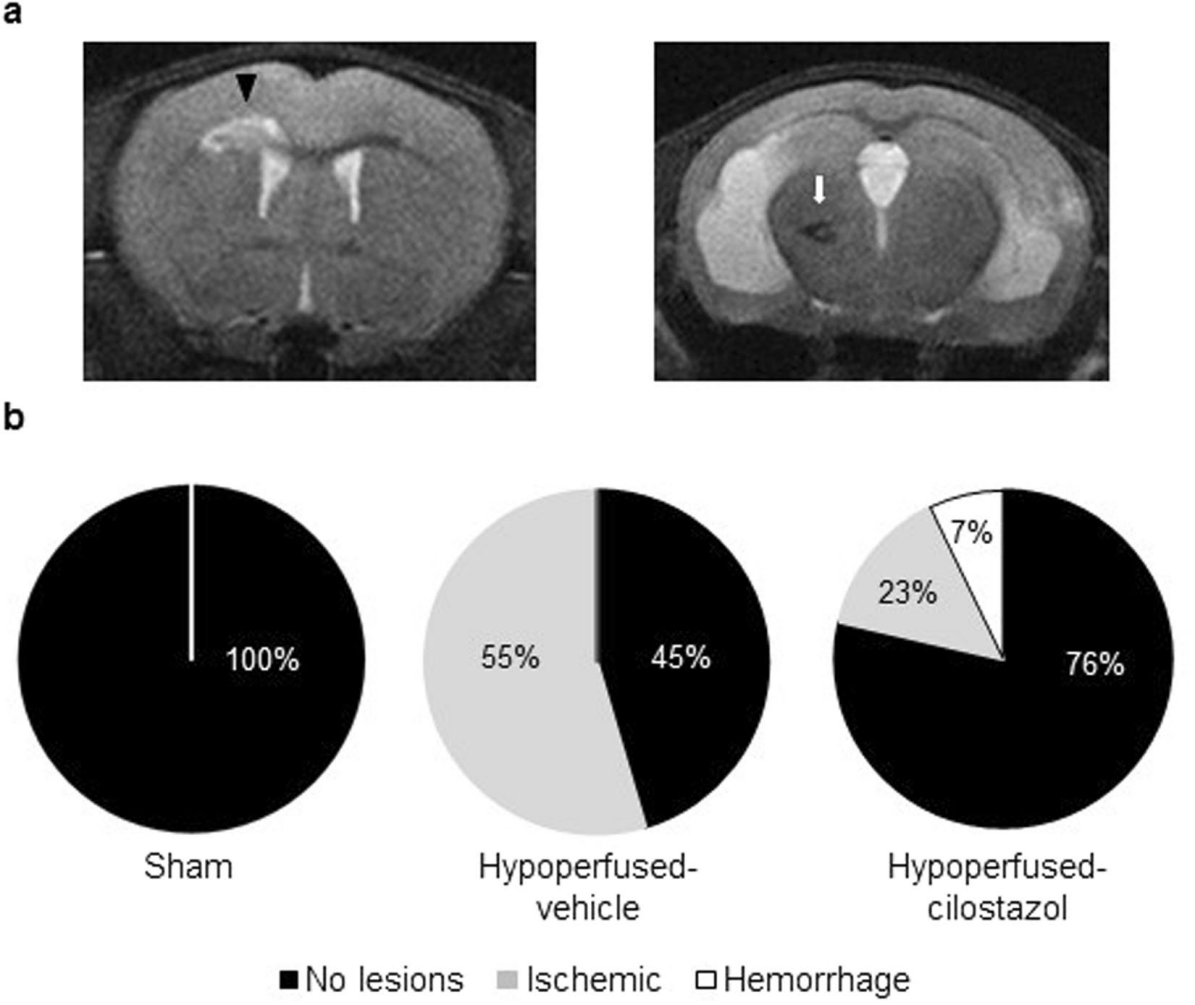
Cilostazol reduced stroke injury. (**a**) Representative images of T2-MRI detecting the ischemic (arrow head) and hemorrhagic (arrow) lesions. (**b**) MRI analysis showed that cilostazol remarkably decreased the number of the ischemic stroke lesions after chronic hypoperfuson.

Following on from the finding that cilostazol had a beneficial effect on endothelial cell activation, we determined whether this may also impact on baseline cerebral blood flow (CBF) using arterial spin labelling (ASL). There was an overall difference in CBF between the three groups in the corpus callosum (*F*_(2, 25)_ = 11.0; *p* = 0.0004) and thalamus (*F*_(2, 25)_ = 33.05; *p* < 0.0001). CBF was significantly reduced in hypoperfused-control and hypoperfused-cilostazol mice compared to sham mice in the corpus callosum (*p* < 0.001 and *p* < 0.001) and the thalamus (*p* < 0.001 and *p* < 0.001) (Fig. 5a and b). Stroke lesions (ischemic and hemorrhagic), detected on T2-MRI (Fig. 6a), were present after hypoperfusion but absent from all sham mice. Interestingly, cilostazol was found to decrease the number of the ischemic stroke lesions (present in only 2 of 13 (15%) hypoperfused-cilostazol mice versus 6 of 11 (55%) hypoperfused-control mice) (Fig. 6b).

## Discussion

The current study is of the first to report the beneficial long-term effects of cilostazol in a model of chronic cerebral hypoperfusion relevant to the pathophysiology of VCI. Cilostazol exerted a protective effect against white matter dysfunction and modestly improved spatial working memory at 3 months after cerebral hypoperfusion. These improved functional outcomes were closely related to reduced gliovascular damage, cellular inflammation and endothelial activation.

**Figure 7:**
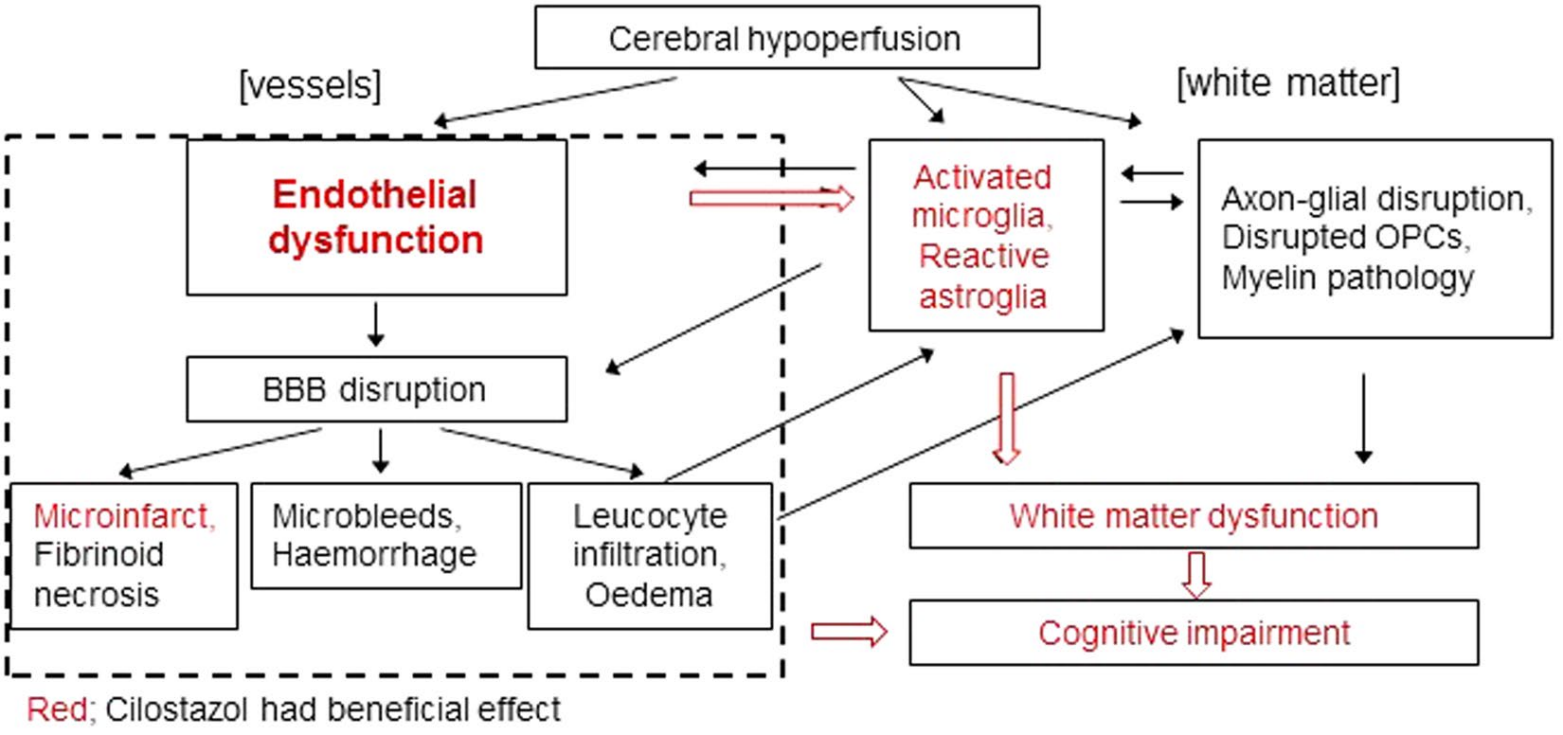
A proposed mechanism by which cilostazol may improve cognitive impairment induced by chronic cerebral hypoperfusion. Cilostazol reduced the number of stroke lesions and significantly ameliorated the spatial working memory impairment and white matter dysfunction induced by chronic cerebral hypoperfusion. Remarkably, cognitive deficits seem to be closely associated with the hypoperfusion-induced neuroinflammatory processes which are significantly decreased with cilostazol treatment. Cilostazol might restore endothelial dysfunction without apparent BBB disruption leading to recovery of neuroinflammation and white matter dysfunction (large arrow).

Previous studies have indicated that cilostazol protects against hypoperfusion-induced white matter damage^13–16^ contrary to the present study in which cilostazol was found to have minimal impact on white matter integrity. The effects of cilostazol have been predominantly studied in more severe models of cerebral hypoperfusion such as a rat 2-vessel occlusion (2VO) model and within 1 to 5 weeks hypoperfusion^13–15^. The rat 2VO model shows marked BBB disruption, as early as 3 days post-operation^22^, leading to prominent white matter lesions, glial activation and profound impairment of various aspects of cognition^15, 23, 24^. In contrast, our mouse model of cerebral hypoperfusion shows gradual and progressive temporal changes of white matter dysfunction, gliovascular unit disruption and glial activation leading to working memory impairment^3, 5^. In our model, there is minimal disruption of the BBB until 6 months post-hypoperfusion^5^ whereas there is marked BBB damage in the rat 2VO models which may enhance movement of cilostazol directly to the brain^25^. There are also differences in the severity of hypoperfusion, BBB and white matter damage between the different laboratories that study the mouse model of cerebral hypoperfusion^16^. We have previously shown that there are subtle changes in the distribution of paranodal-nodal proteins with hypoperfusion^4^ that were not measured in the present study. This redistribution of the molecular architecture of myelinated axons can impact on white matter function. In support of this, despite the lack of protection against white matter changes, cilostazol was able to improve white matter function as assessed using electrophysiological approaches and improve spatial working memory which is dependent on frontal cortical circuitry. Increased microglial density accompany hypoperfusion-induced damage to white matter^3–5, 15, 16^ and may contribute to the damage via pro-inflammatory mechanisms. Notably increased microglia were determined in the corpus callosum of hypoperfused mice and reduced by cilostazol-treatment. Furthermore, it was found that the extent of microgliosis in white matter relates to the impairment in working memory. A previous study has also shown beneficial effects of cilostazol on microglia and anti-inflammatory pathways^26^. Thus neuroinflammation is proposed to be strongly associated with white matter dysfunction and working memory impairment (Fig. 7).

Endothelial dysfunction is widely considered to be one of the pivotal mechanisms of the structural and functional brain-vessel alterations in small vessel disease (SVD) and evidence has accumulated to support the hypothesis that early endothelial failure is a major precipitant of sporadic SVD^27, 28^. Endothelial damage can lead to increased permeability, damage to vessel wall, dysregulation of vascular tone, inflammation, demyelination, gliosis and at a late stage, luminal narrowing and thrombosis^21, 27^. White matter lesions are characterized by the expression of adhesion molecules such as ICAM1^28^, which are induced by proinflammatory cytokines that promote the adherence of monocytes. The increased expression of ICAM1 may reflect endothelial dysfunction or microvascular inflammation; ICAM1 expression is recognized mainly in the endothelial cells but can also be found in microglia and leukocytes. In the current study, ICAM1 staining, as an index of endothelial dysfunction, was markedly increased with hypoperfusion and restored with cilostazol treatment. Additionally endothelial dysfunction was significantly correlated with neuroinflammation and spatial working memory impairment. Thus the data suggest that cilostazol might restore endothelial dysfunction without apparent BBB disruption leading to dampening of glial activation, improved white matter function and cognition (Fig. 7, large arrows). However whether this is a direct effect of cilostazol on endothelial cells or secondary to other effects, for example on microglia^26^, remains to be determined. Interestingly cilostazol has been shown to have effects on spatial learning in control mice via effects on growth factors^29^. Thus cilostazol appears to have multiple beneficial effects. Future studies could define whether endothelium dependent vasodilator agents afford similar protection in the hypoperfusion model *in vivo* in comparison to cilostazol treatment. Similarly, in animal models relevant to Alzheimer’s disease (AD), increased endothelial adhesion molecules have been highlighted as markers of endothelial dysfunction and vascular inflammation^30^. Endothelial dysfunction may be a common target in the treatment of AD, VCI and vascular dementia. Our present study supports the utility of cilostazol in the treatment of VCI. A previous study has also indicated beneficial effects of cilostazol in a mouse model relevant to AD through increased amyloid β clearance due to improved cerebrovascular function^31^. Retrospective clinical studies indicated the potential for cilostazol treatment to suppress cognitive decline in elderly patients with mild dementia or cognitive impairment^32, 33^. However the current study suggests that whilst cilostazol improves hypoperfusion- induced memory deficits these are not completely restored, the improvement in memory plateaued after 12 days of testing. The current studies were also conducted in mid-age rodents and further studies should include aged cohorts where the pathological and functional impairments to hypoperfusion may be exacerbated and be affected differently by cilostazol.

Although cilostazol is well known for its vasodilatory effect via up-regulation of cAMP, there was no effect of cilostazol determined on CBF measures in the corpus callosum and the thalamus at 3 months post-operation. These results are consistent with other studies^13, 14^, which indicated that CBF was not significantly influenced by cilostazol despite demonstrations that white matter damage is restored in the cilostazol treated mice^14^. Similarly, although there was a reduction in the number of stroke lesions with cilostazol this was not paralleled by an increase in CBF. In this study it is difficult to precisely relate the CBF measures with stroke lesions since the CBF measures using ASL were conducted at one brain level whereas the stroke lesions were found throughout different levels of the brain and in different brain regions. There may be other effects of cilostazol that are independent of perfusion. For example, restoration of endothelial dysfunction by cilostazol directly or indirectly via microglia^33^ might reduce the number of stroke lesions without an effect on perfusion (Fig. 7). Vasomotor reactivity or neurovascular coupling, which are recognized to be associated with cognitive impairment^34, 35^ might also be impaired by hypoperfusion and remain to be elucidated.

Our study showed that cilostazol ameliorated gliovascular damage and working memory impairment possibly via endothelial protection in a mouse model of chronic cerebral hypoperfusion. This evidence indicates that cilostazol, originally a drug for stroke and peripheral arterial disease, might be a useful candidate for the treatment of VCI. However, these beneficial effects of cilostazol are modest in this model of VCI and thus other more potent drugs need to be considered.

## Methods

### Animals and surgical procedure

All experiments were conducted under the UK Home Office Animals (Scientific Procedures).

Act 1986, in agreement with local ethical and veterinary approval (Biomedical Research Resources, University of Edinburgh) and the ARRIVE guidelines. Adult (25–30 g, approx. 4–5 months old) male C57Bl/6J mice (Charles River Laboratories Inc., UK) were used for all experiments. Mice underwent chronic cerebral hypoperfusion via bilateral common carotid artery stenosis^3–5^ using microcoils fitted on both common carotid arteries under isoflurane anaesthesia (induced at 5% and maintained at 1.5%). Sham mice underwent the exact same surgical procedure with the exception that coils were not applied to the arteries. Mice were fed with either pelleted chow containing 0.3% cilostazol (cilostazol-treated mice) or standard pelleted chow only (control-treated mice) from one week before the surgery.

### Animal cohorts and exclusions

Mice were coded and randomly divided into different groups (1) sham-control, (2) hypoperfused-control, and (3) hypoperfused-cilostazol. Two different cohorts were studied: **Cohort 1** in which the mice were assessed using Radial Arm Maze, Magnetic Resonance Imaging (MRI) with endpoints of pathology; **Cohort 2**; mice were studied using electrophysiology. Investigators were blinded to the surgical or drug intervention until the final analysis. **Cohort 1** Initial numbers at the outset of study were: sham-control n = 10, hypoperfused-control n = 12, hypoperfused-cilostazol n = 13. One mouse in the hypoperfused-control group was culled due to poor recovery before the MRI scan. The final numbers for behaviour, were sham-control n = 10, hypoperfused-control n = 12, hypoperfused-cilostazol n = 13. The final numbers for MRI (T2/DTI) were sham-control n = 10, hypoperfused-control n = 11, hypoperfused-cilostazol n = 13. ASL was assessed in a subset of these since one mouse in sham-control, four mice in hypoperfused-control and three mice in hypoperfused-cilostazol group were excluded due to poor quality of ASL. Thus the final group sizes for ASL were: sham-control n = 9, hypoperfused-control n = 7, hypoperfused-cilostazol n = 10. **Cohort 2**: Initial numbers at the outset were sham-control n = 4, hypoperfused-control n = 6, hypoperfused-cilostazol n = 5. Electrophysiology recordings could not be made in two mice: one hypoperfused control and one hypoperfused cilostazol. Thus the final numbers for electrophysiology were sham-control n = 4, hypoperfused-control n = 5, hypoperfused-cilostazol n = 4.

### Radial Arm maze

Spatial working memory was assessed using an 8-arm radial maze. Mice were food deprived to reduce their initial body weight by 10–15% and the restricted feeding was maintained until the end of behavioural testing. Two pretraining days were undertaken in order to familiarize the animals with the experimental environment, the apparatus and the task itself (see supplementary methods). For the training procedure one food pellet was placed at the end of each of the 8 arms of the maze. At the beginning of every trial (one trial/day) the animal was placed on the central platform with all arm doors open and allowed to make an arm choice. An arm choice was recorded when the centre of the mouse (as tracked by the *AnyMaze* software) was 5 cm into the arm. Once the mouse had entered one of the arms, the doors to the other 7 arms were closed automatically. When the animal exited the visited arm it was confined on the central platform for 5 sec by closing the remaining door. After the 5 sec delay it was allowed to make a new choice. A trial ended when the mouse had retrieved all 8 pellets or 25 min had elapsed. The behavioural testing lasted 16 days. For each trial, the number of correct arm entries within the first eight visits, the number of revisited arms (working memory errors) and the time taken to complete the task were recorded. Data were expressed as the mean of 4 trial blocks for the first 8 days and 2 trial blocks for the rest of 8 days.

### Magnetic Resonance Imaging

Neuroimaging was conducted on completion of the behavioural assessment. Mice were anaesthetised and placed in an MRI compatible holder (Rapid Biomedical, Wurzburg, Germany). Rectal temperature and respiration were monitored and controlled throughout to ensure normal physiological parameters. Structural T2-weighted and DT- MRI data were collected using an Agilent 7 T preclinical MRI scanner (Agilent Technologies, Yarnton, UK); with a 72 mm volume coil and a 2 channel phased array mouse brain coil (Rapid Biomedical). Arterial Spin Labelling (ASL) was performed using a Look-Locker FAIR single gradient echo (LLFAIRGE) sequence (see supplementary methods for details).

T2-weighted MRI slices of the mouse brain (1.0 to − 4.6 mm Bregma) were examined for (i) the presence and type of cortical and/or subcortical primary ischaemic lesions and (ii) primary haemorrhages and anatomical location^3^ (supplementary methods). The number of ischaemic and hemorrhagic lesions were determined and the percentage of mice with lesions in each cohort calculated. Fractional Anisotropy (FA) was generated using ‘in-house’ custom software. FA and ASL techniques were processed by regions of interest (ROIs) analysis. ROIs were set in the thalamus and the corpus callosum at the levels of the striatum and the hippocampus for FA and at the level of hippocampus for ASL.

### Immunohistochemistry

Mice were transcardially perfused with 4% paraformaldehyde (PFA) under deep anaesthesia (5% isoflurane in oxygen enriched air) and brains were carefully removed, postfixed in 4% PFA for 24 hours and processed for cryostat sectioning. Coronal brain sections were cut using a cryostat and mounted onto superfrost slides (10 µm) or preserved in cryoprotective medium at −20 °C (30 µm). All sections except for ICAM1 staining were heat-retrieved with either EDTA pH 8 or citrate pH 6 and primary antibodies were incubated overnight at 4 °C in blocking buffer (see supplementary data for further details). Subsequently, the sections were incubated with biotinylated secondary antibodies (1:100; Vector Labs) and a solution of streptavidinbiotin-peroxidase complex or alternatively with fluorescent secondary antibodies (Alexa Fluor, Thermofisher; 1:500). Peroxidase activity was localised using 3,3′ diaminobenzadine tetrahydrochloride (DAB) as a chromagenic substrate (Vector Labs). DAB immunolabeled sections were analyzed using an optical microscope Olympus BX51 and fluorescent immunolabeled sections were analyzed using a laser scanning confocal microscope Leica SP5 C. All measurements were done in the corpus callosum using imageJ. To assess microglial activation, expression of endothelial adhesion molecule and activation of astrocytes, positively stained area of Iba1, ICAM1 and GFAP were measured respectively. The loss of axon-glial integrity of the corpus callosum (disorganised white matter fibres, presence of myelin debris and vacuolation) was assessed using MAG antibody and graded from 0 (none) to 3 (extensive) as previously described^3^. Percentage area stained by AQP4 and COL4 as well as their respective colocalization (Manders coefficient) was determined after being equally thresholded across all sections for the respective antibody. The measure of AQP4 out with vessels was defined as the difference between percentage area stained by AQP4 and COL4, respectively.

### Electrophysiology

Three months after surgery, mice were sacrificed and their brains were quickly removed on top of a frozen petri-dish with a few drops of sucrose artificial cerebrospinal fluid (aCSF) (189 mM Sucrose, 10 mM D-glucose, 26 mM NaHCO3, 3 mM KCl, 5 mM MgCl2, 0.1 mM CaCl2, 1.25 mM NaH2PO4). The brain was then rapidly dissected, placed in a cell strainer and submerged in ice-cold oxygenated sucrose aCSF for 2–3 minutes. Sections were then cut at 400 μm using a vibrating blade microtome (Hydrax V50, Zeiss, Cambridge, UK). A single coronal slice (−1.2–1.7 mm Bregma) was then transferred to a warmed (34 °C) incubation chamber (BSK1, Digitimer, Welwyn Garden City, UK) with oxygenated aCSF (124 mM NaCl, 5 mM KCL, 1.25 mM NaH_2_PO_4_, 26 mM NaHCO_3_, 1.3 mM MgSO_4_, 2 mM CaCl_2_, 10 mM D-Glucose). Following at least a 1 hour period of post-slicing recovery slices were carefully transferred to a recording chamber (RC27L, Harvard Apparatus, Cambridge, UK) and mounted on an Olympus BX51 microscope where they were superfused with oxygenated aCSF (2–3 ml/min) at 24 °C. This temperature was employed to enable discrimination of compound actions potentials (CAP) arising from myelinated and unmyelinated fibres. Slices were supported by a slice anchor and then allowed to rest for 30 minutes prior to recording. A bipolar stimulating electrode (WPI Inc, Hertfordshire, UK) with manually blunted tips was lowered into the corpus callosum ~1 mm lateral to the midline using a manual manipulator and constant current stimulus-isolated square wave pulses (NL800, Digitimer, Welwyn Garden City, UK) were applied to evoke CAPs. Recording electrodes were pulled from borosilicate glass capillaries (1.5/0.84 mm OD/ID, 100 mm long, WPI Inc., Hertfordshire, UK) and back filled with aCSF to give a final resistance of 1–3 MΩ. These were connected to the amplifier’s headstage via an Ag/AgCl wire and then lowered into the corpus callosum (Patchstar micromanipulator, Scientifica, East Sussex, UK) adjacent to the midline 2.5 mm contralateral to the stimulating electrode. For analysis of the CAP peak amplitude standardised input-output functions were generated by varying the intensity of stimulus pulse (200 μs duration, 0.2 Hz) in 0.25 mA increments from 0.25–4 mA. To enhance the signal to noise ratio, all electrophysiology analysis was conducted on averaged waveforms of four successive sweeps. Evoked CAPs were amplified (x500) and filtered (bandpass = DC to 10 kHz) using an Axopatch 200 A amplifier in voltage following mode (Molecular Devices, Berkshire, UK). Responses were digitized at 200 kHz, and recorded for offline analysis using pClamp software (v10, Molecular Devices, Berkshire, UK). CAPs were evoked at the maximal stimulation of 4 mA and the distance between the stimulating and recording electrode altered in 0.5 mm increments from 1–2.5 mm by moving the recording electrode. The post-stimulus latency of the CAP peak was then measured.

### Statistical analysis

All values are expressed as mean ± standard error of the mean (SEM). One-way analysis of variance (ANOVA) was used to evaluate significant differences among groups followed by a post hoc Tukey test. When data was not normally distributed, non-parametric tests were applied (Kruskal-Wallis). Semi-qualitative MAG grading was analysed with Kruskal-Wallis test for trend. Spearman’s or Pearson’s rho was used to evaluate the correlation betweeen data sets. Differences with *p* < 0.05 were considered significant in all statistical analyses.

## Electronic supplementary material

Supplementary
