# Supplementary material for: Long-term cilostazol treatment reduces gliovascular damage and memory impairment in a mouse model of chronic cerebral hypoperfusion
Source: Sci Rep. 2017 Jun 27;7:4299. doi: 10.1038/s41598-017-04082-0 (PMC5487324; doi:10.1038/s41598-017-04082-0)
Supplement: Supplementary file 1 — Supplementary [file 41598_2017_4082_MOESM1_ESM.doc]

**Long-term cilostazol treatment reduces gliovascular damage and memory impairment in a mouse model of chronic cerebral hypoperfusion**

Akihiro Kitamura MD, PhD^1#^, Yasmina Manso PhD^1#·^, Jessica Duncombe BSc (Hons)^1^, James Searcy PhD^1^, Juraj Koudelka PhD^1^, Margaret Binnie BSc (Hons) ^1^, Scott Webster PhD^1^, Ross Lennen B.Sc^1^, Maurits Jansen PhD^1^, Ian Marshall PhD^1^, Masafumi Ihara MD, PhD^2^, Raj N. Kalaria MD,PhD ^3^, Karen Horsburgh PhD^1*^

**Supplementary Methods**

**Animal cohorts and exclusions**

**Cohort 1** Initial numbers at the outset of study were: sham-control n=10, hypoperfused-control n=12, hypoperfused-cilostazol n=13. One mouse in the hypoperfused-control group was culled due to poor recovery before the MRI scan.  The final numbers for behaviour, were sham-control n=10, hypoperfused-control n=12, hypoperfused-cilostazol n=13. The final numbers for MRI (T2/DTI) were sham-control n=10, hypoperfused-control n=11, hypoperfused-cilostazol n=13. ASL was assessed in a subset of these since one mouse in sham-control, four mice in hypoperfused-control and three mice in hypoperfused-cilostazol group were excluded due to poor quality of ASL. Thus the final group sizes for ASL were:  sham-control n=9, hypoperfused-control n=7, hypoperfused-cilostazol n=10. **Cohort 2**: Initial numbers at the outset were sham-control n=4, hypoperfused-control n=6, hypoperfused-cilostazol n=5. Electrophysiology recordings could not be made in two mice: one hypoperfused-control and one hypoperfused cilostazol. Thus the final numbers for electrophysiology were sham-control n=4, hypoperfused-control n=5, hypoperfused-cilostazol n=4. Note these numbers were based on our previous experience of the variation of peak latency in a hypoperfused and control group with a SD of 15% and predicted effect size of 20% at 80% power and p<0.05.

**Assessment of spatial working memory using a radial arm maze test**

Behavioural testing took place on an 8-arm radial maze made of white plastic with transparent Plexiglas arm walls (20 cm tall). The maze consisted of an octagonal central platform (20 cm diameter) with 8 equally spaced arms radiating from it (47 cm long, 7 cm wide). At the distal end of each arm, a plastic food well (3.5 cm diameter, 2 cm deep) was located. Doors, located at the proximal end of each arm, were controlled remotely using Any-Maze software (Stoelting, UK), which also recorded the behaviour and allowed for automation of door opening and closing based on tracking of each animal’s position in the maze. The maze was elevated 1 m from the floor of the experimental room. A camera connected to a PC was fixed on the ceiling just above the central platform of the maze. The experimental room was dimly lit, and equipped with salient extra maze cues. On pretraining day one, food pellets (45mg, Bio-Serv) were scattered around the maze and each animal was left to explore freely the maze for 5 min. On pretraining day 2, a single food pellet was placed at the end of each of the eight arms. The mouse was placed in the central platform and allowed access to each arm in turn (controlled using the doors), and allowed to retrieve the food pellet.

**Magnetic Resonance Imaging**

Structural T2-weighted and DT- MRI data were collected using an Agilent 7T preclinical MRI scanner (Agilent Technologies, Yarnton, UK); with a 72 mm volume coil and a 2 channel phased array mouse brain coil (Rapid Biomedical). Fifteen contiguous slice locations were imaged using a T2-weighted fast spin echo structural sequence with a field-of-view (FOV) of 19.5 × 19.5 mm, an acquisition matrix of 192 × 192 and slice thickness of 0.8mm, giving an acquisition voxel dimension of 0.1 × 0.1 × 0.8 mm. The repetition (TR) and echo (TE) times were 3000 and 36ms respectively. The diffusion tensor (DT-)MRI protocol consisted of 10 T2-weighted and sets of diffusion-weighted (b=1000s/mm^2^) axial fast spin-echo volumes acquired with diffusion gradients applied in 60 non-collinear directions, producing a total of 70 volumes.[^14^](#_ENREF_3) Fifteen slice locations identical to those used in the T2-weighted scan were imaged with a FOV of 19.5 × 19.5mm, an acquisition matrix of 96 × 96 (zero filled to 128 × 128), giving an acquisition voxel dimension of 0.2 × 0.2 × 0.8 mm. The TR and TE times for each fast spin-echo volume were 2000 and 35ms respectively. In all the slices analyzed there was minimal disruption to the MR signal caused by the presence of the microcoils and all brain regions analyzed were unaffected. T2-weighted MRI slices of the mouse brain (1.0 to − 4.6 mm Bregma) were examined for (i) the presence and type of cortical and/or subcortical primary ischaemic lesions, classified as hyperintense signal with focal tissue loss, potentially cavitated, with or without localized hypointense areas consistent with paramagnetic effects of iron/haemosiderin in the surrounding parenchyma; and (ii) primary haemorrhages and anatomical location, classified as areas of rounded predominantly hypointense signal[^3^](#_ENREF_33).

ASL was performed using a Look-Locker FAIR single gradient echo (LLFAIRGE) sequence. Forty gradient echoes spaced 200ms apart were acquired after a slice-selective or global adiabatic inversion pulse for each phase-encoding, resulting in a total observation time of approximately 16 minutes for a 64×64 imaging matrix. The flip angle was 20˚. The first 20˚ pulse occurred 3 ms after the inversion pulse. The echo time was 1.42 ms.

**Investigation of gliovascular unit using immunohistochemistry**

Ten μm-thick sections mounted on superfrost slides were incubated overnight with ionized calcium binding adaptor molecule 1 antibody (Iba1; 1:1000 for DAB, Menarini), myelin-associated glycoprotein antibody (MAG; 1:500 for DAB, Santa Cruz Biotechnology) and intercellular adhesion molecule 1 antibody (ICAM-1; 1:500 for DAB, R & D Systems). To assess microglial activation and expression of endothelial adhesion molecule, positively stained area of Iba1 and ICAM1 were measured (mean±SEM %). The loss of axon-glial integrity of the corpus callosum (disorganised WM fibres and myelin debris) was assessed using MAG antibody and graded from 0 (none) to 3 (extensive); normal (grade 0), minimal myelin debris, vacuolation and disorganisation of fibres (grade 1), modest myelin debris, vacuolation and disorganisation of fibres (grade 2), and extensive myelin debris, vacuolation and disorganisation of fibres (grade 3) [^2^](#_ENREF_2). 30-μm-thick sections mounted on superfrost slides were incubated overnight with glial fibrillary acidic protein antibody (GFAP; 1:100, Life Technologies), aquaporin 4 antibody (AQP4; 1:100, Millipore) and COL4 antibody (1:100, Millipore) and subsequently incubated for one hour at room temperature with fluorescent secondary antibodies Alexa Fluor 488, 546 and 647 (1:500) and Streptavidin Alexa Fluor 546 (1:100). Sections were analyzed using a laser scanning confocal microscope Leica SP5 C (Milton Keynes, UK).
